# Hypo-phosphorylated CD147 promotes migration and invasion of hepatocellular carcinoma cells and predicts a poor prognosis

**DOI:** 10.1007/s13402-019-00444-0

**Published:** 2019-04-23

**Authors:** Jin Jin, Shi-Jie Wang, Jian Cui, Ling Li, Jia-Yue Li, Fen-Ling Liu, Xiu-Xuan Sun, Jian-Li Jiang, Hong-Yong Cui, Zhi-Nan Chen

**Affiliations:** 1 Department of Cell Biology, National Translational Science Center for Molecular Medicine, Fourth Military Medical University, Xi’an, 710032 People’s Republic of China; 2 State Key Laboratory of Pharmaceutical Biotechnology, Department of Biotechnology and Pharmaceutical Sciences, School of Life Sciences, Nanjing University, Nanjing, 210023 People’s Republic of China

**Keywords:** Hepatocellular carcinoma, CD147, Phosphorylation, Invasion, Metastasis

## Abstract

**Purpose:**

CD147 is a tumor-associated antigen that plays a key regulatory role in tumor invasion and distant metastasis. However, the exact role of CD147 phosphorylation, which is deregulated during cancer progression, is unknown. Here, the effects of CD147 phosphorylation on the malignant behavior of hepatocellular carcinoma (HCC) cells and its possible underlying mechanisms are explored.

**Methods:**

An in situ Duolink-proximity ligation assay (PLA) was used to detect CD147 phosphorylation. Tandem mass spectrometry was employed to identify the phosphorylation sites of CD147. The effects of CD147 phosphorylation on the malignant behavior of HCC cells were evaluated using scratch wound healing assays, transwell invasion assays and cell cycle assays. The genes regulated by CD147 phosphorylation were detected by RNA sequencing.

**Results:**

We identified phosphorylated serine-246 in the C terminus of CD147 in primary HCC tissues, whereas serine to alanine substitution mutation analysis suggested that CD147 is phosphorylated mainly at serine-252 in HCC-derived Huh-7 cells. Recovery expression of S246A/S252A mutants in CD147 knockout cells revealed significantly increased migration and invasion capacities compared to wildtype CD147 expressing cells. Cyclophilin A (CyPA) treatment decreased the phosphorylation level of CD147, whereas NIMA-related kinase 6 (NEK6) increased the CD147 phosphorylation level. Moreover, the CD147 phosphorylation level was found to be dramatically decreased in HCC tissues in patients with distant metastases, and a low phosphorylation level of CD147 was found to be associated with a high serum AFP level, recurrence and a poor overall survival.

**Conclusions:**

From our data we conclude that hypo-phosphorylated CD147 promotes the migration and invasion of HCC cells and correlates with an unfavorable prognosis in HCC patients, indicating that targeting the aberrantly hypo-phosphorylated form of CD147 may be instrumental for the development of novel therapeutic modalities directed against HCC metastasis.

**Electronic supplementary material:**

The online version of this article (10.1007/s13402-019-00444-0) contains supplementary material, which is available to authorized users.

## Introduction

Hepatocellular carcinoma (HCC) is one of the most common malignancies and one of the most frequent causes of cancer-related death in the world [1]. Despite significant improvements in both diagnostic and therapeutic modalities, metastasis is still a major contributor of treatment failure and death [2–4]. The capacity of cancer cells to metastasize to distant sites is controlled by complicated cellular processes involving microenvironmental changes and increasing cell migration and invasion abilities, as well as multiple genetic events and alterations in regulatory factors [5]. Nevertheless, despite the progress that has been made in elucidating the molecular mechanisms underlying HCC metastasis [6, 7], its relapse rates remain high after resection, and the relapses nearly always originate from metastases [8]. Failures to combat HCC invasion and metastasis have become major obstacles to improvements in the survival and quality of life of HCC patients [9].

CD147, also known as extracellular matrix metalloproteinase inducer (EMMPRIN), is a multifunctional cell adhesion molecule that plays important roles in both normal physiological and pathological conditions, including reproduction, development, immunological responses, infectious diseases and malignant tumors [10]. CD147 has been found to be overexpressed in a broad range of human malignant tumors including HCC [11] and has been implicated in various aspects of tumor progression, in particular HCC metastasis [12]. We and others have previously shown that CD147 can promote the migration, invasion, proliferation and survival of tumor cells [13–17]. In addition, overexpression of CD147 in tumor cells as well as in serum has been recognized as an unfavourable prognostic factor [18, 19].

CD147 functions through multiple molecular mechanisms. CD147 is best known as a potent inducer of extracellular matrix metalloproteinases (MMPs) and has been found to induce the production of MMPs by tumor cells as well as mesenchymal cells [20]. CD147 has also been found to be involved in epithelial mesenchymal transition (EMT), cytoskeleton rearrangement and the formation of lamellipodia and invadopodia [21, 22]. CD147 serves as a receptor for several molecules through trans-recognition using its extracellular Ig domain [10]. A typical ligand for CD147 is Cyclophilin A (CyPA). CyPA is secreted by endothelial cells within the bone marrow and attracts myeloma cells that strongly express CD147 [23]. As yet, however, it is far from clear how, upon receiving a signal through trans-recognition, this signal is transduced and whether the intracellular domain of CD147 is participating in this process.

Post-translational modifications (PTMs) such as glycosylation and phosphorylation can fine-tune the cellular functions of proteins. Uncovering the relationships between PTMs and functional changes is critical for our understanding of the molecular mechanisms underlying particular cellular processes. Previously, we found that CD147 purified from human lung cancer tissue was N-glycosylated and contained a series of high-mannose and complex-type N-linked glycan structures. Subsequent mutation analysis revealed that N-glycosylation was crucial for CD147 protein folding and MMP induction [24]. Protein phosphorylation plays a central role in cellular signaling and is employed by cells to transiently alter protein localization, conformation and interaction with other proteins. When deregulated, it may also be involved in disease processes, notably cancer. Phosphoproteome analysis has revealed that CD147 can be phosphorylated at Ser246 and/or Ser252 in various human tissues and its derived cell lines, including those from muscle [25], liver [26, 27], B cell non-Hodgkin lymphoma [28] and lung cancer [29], indicating that phosphorylation is an important form of CD147 post translational modification. The function of CD147 phosphorylation in both normal physiological and pathological conditions is as yet, however, unknown.

Here, we show that CD147 is phosphorylated in primary HCC tissues and derived cell lines, with major phosphorylation sites at S246 and S252 in HCC tissues and Huh-7 cells, respectively. Abolishing CD147 phosphorylation by mutating S246 and S252 (S246A/S252A) led to expression alteration of a set of genes related to extracellular matrix (ECM) remodeling and cell migration and invasion enhancement via STAT3 and Akt signaling. Moreover, we found that the phosphorylation level of CD147 was dramatically decreased in HCCs with distant metastases and that low CD147 phosphorylation levels were associated with high serum AFP levels, disease recurrence and a poor overall survival, suggesting that the aberrantly hypo-phosphorylated form of CD147 may serve as a valuable biomarker for prognosis assessment and the development of novel therapeutic modalities directed against HCC metastasis.

## Materials and methods

### Reagents

TRIzol was purchased from Sigma (St Louis, Missouri, USA). Antibodies directed against phospho-serine (ab9392) and α-tubulin (ab80779) were purchased from Abcam (Cambridge, MA, USA); Antibodies directed against HA tag (0906–1), STAT3 (ET1605–45), p-STAT3 (ET1603–40), Akt (ET1609–47), p-Akt (ET1607–73), c-Jun (ET1608–3), p-c-Jun (ET1608–4) and p38 (ET1602–26) were obtain from HuaAn Biotechnology (Hangzhou, China); goat anti-mouse IgG antibody (31430), goat anti‐rabbit IgG antibody (31460), anti‐p‐p38 antibody (44‐684G) and anti‐NEK6 antibody (MA5–24947) were purchased from Thermo Fisher Scientifc (Waltham, MA, USA); CD147-specific antibody was produced by our lab.

### Cell lines and culture conditions

Human hepatocellular carcinoma (HCC) cell line Huh-7 was obtained from the Japanese Collection of Research Bioresources (JCRB, Osaka, Japan). HepG2 and SMMC-7721 cells were obtained from the Chinese Academy of Medical Sciences (Shanghai, China). A Huh-7 CD147-KO (Huh-7 CD147^−/−^) cell line was generated using a CRISPR/Cas9 system as previously reported [14]. All cells were cultured at 37 °C, 5% CO_2_, in RPMI-1640 medium supplemented with 10% fetal bovine serum (FBS). All cells have been authenticated using short tandem repeat profiling.

### Tissue specimens and immunocytochemistry

76 HCC tissue specimens were collected from the Department of Pathology, Eastern Hepatobiliary Surgery Hospital, which is affiliated with the Second Military Medical University, from 2008 to 2012 and were histological confirmed by staining with hematoxylin and eosin (HE). All patients provided written informed consent, and the study was approved by the hospital Ethics Committee

Immunohistochemical (IHC) staining was performed on 5 μm tissue sections. To this end, paraffin sections were dewaxed, followed by antigen retrieval with 10 μM citrate buffer at pH 6.0. The deparaffinized sections were treated with methanol containing 3% hydrogen peroxide for 15 min. After washing with PBS, the sections were incubated with blocking serum for 30 min. Then, the sections were incubated with anti-CD147 antibody at 4 °C overnight. Following incubation, immunoperoxidase staining was conducted using a streptavidin-peroxidase kit (Zhongshan Jinqiao Co., Beijing, China) and the sections were treated with 3,3′-diaminobenzidine (Zhongshan Jinqiao Co., Beijing, China) to detect the target proteins. Hematoxylin was used to counterstain the nuclei. The expression levels were independently evaluated by two senior pathologists according to the proportion and intensity of positive cells. The following criteria were used to score each specimen: 0 (no staining), 1 (any percentage with weak intensity or < 30% with intermediate intensity), 2 (> 30% with intermediate intensity or < 50% with strong intensity) or 3 (> 50% with strong intensity).

### Immunofluorescence assay

Immunofluorescence was performed as described previously [13]. Briefly, cells were harvested and allowed to attach for 24 h to fibronectin pre-coated cell culture dishes with glass bottoms (NEST Biotechnology Co., LTD.). After washing twice with PBS, the cells were fixed with paraformaldehyde in PBS, permeabilized with 0.1% Triton X-100, and blocked with 1% BSA in PBS for 1 h. The resulting cells were first incubated with the indicated antibodies for 1 h, washed twice with PBS, and then incubated with Alexa 488-phalloidin solution and the corresponding FITC-conjugated secondary antibodies for 30 min in the dark. Cell nuclei were stained with DAPI (Vector Labs). After washing, the cells were visualized using an A1R-A1 confocal laser microscope (Nikon, Japan).

### In situ proximity ligation assay

In situ proximity ligation assay (PLA) experiments were performed using reagents and instructions provided by a commercially available kit (Duolink In Situ Detection Reagents Red) from Sigma-Aldrich (St Louis, MI, USA). Briefly, cells were seeded into dishes with glass bottoms (NEST Biotechnology Co., LTD.). After washing twice with PBS, the cells were fixed in paraformaldehyde for 15 min at room temperature and blocked with Blocking Solution for 1 h at 37 °C. Next, the cells were rinsed twice with PBS/0.1% Tween 20 (PBST) after which primary mouse anti-CD147 (HAb18, prepared by our laboratory, 1:1000) and rabbit anti-phospho-serine (Abcam, ab9392, 1:100) antibodies in Duolink In Situ Antibody Diluent were applied and incubated overnight at 4 °C. Next, the cells were rinsed three times with Wash Buffer A. Secondary probes (anti-mouse-PLUS and anti-rabbit-MINUS, conjugated to oligonucleotides) were diluted to final concentrations of 1:5 in antibody diluent. The secondary probe mix was added to each sample, incubated for 1 h at 37 °C and washed with Wash Buffer A, after which 40 μl ligation solution was added. The dishes were incubated for 30 min at 37 °C. After washing with Wash Buffer A, 40 μl amplification solution was added and incubated for 100 min at 37 °C. Next, the cells were rinsed three times with Wash Buffer B. Before detection, 50 μl Duolink In Situ Mounting Medium with DAPI was added to each sample. Images were captured using a fluorescence microscope, and PLA signals were analyzed using the Duolink ImageTool software (Sigma-Aldrich). Formalin-fixed paraffin-embedded (FFPE) HCC tissue samples were prepared as described under 2.3 prior to the blocking step. Next, the assay was performed according to the manufacturer’s protocol.

### Transfection and generation of stable cell lines

One day prior to transfection, 4 × 10^5^ cells were seeded per well in a 12-well plate in complete medium. Subsequent transfection was carried out using Lipofectamine 2000 (Invitrogen, Carlsbad, CA, USA) according to the manufacturer’s instructions. After transfection, the cells were subjected to selection in 100 μg/ml G418 for 2 weeks. Antibiotic resistant colonies were subsequently picked, pooled, and expanded for further analysis under selective conditions.

### Co-immunoprecipitation assay

Co-immunoprecipitation (co-IP) was performed using a Pierce™ Co-IP Kit (Thermo Fisher Scientifc, MA, USA) according to the manufacturer’s protocol. For each co-IP assay 10 μg affinity-purified antibody was used. Cell lysates were incubated with gentle rocking overnight at 4 °C. The eluted samples were analyzed by Western blotting using antibodies as indicated.

### Western blotting

Western blotting was performed as described previously [13]. Briefly, equal amounts of protein were separated by denaturing SDS-PAGE and transfered to polyvinylidene fluoride (PVDF) microporous membranes (Millipore, Boston, MA). Next, the resulting blots were blocked with 5% nonfat milk in TBS/0.5‰ Tween (TBS-T). The primary antibodies were diluted in TBS-T, and the blots were incubated with these antibodies overnight at 4 °C followed by washing in TBS-T and incubation with HRP-conjugated secondary antibodies for 1 h at room temperature. Signal detection was conducted using a ChemiDoc™ Touch Imaging System and analyzed using Image Lab™ Software (Bio-Rad, CA, USA).

### Scratch wound healing assay

In vitro scratch wound healing assays were performed as described previously [13]. Briefly, 24 h after treatment, the cells were harvested, seeded in 12-well plates and grown until confluence. Next, a pipette was used to scratch (‘wound’) the monolayer after which the remaining cells were washed with serum-free medium. Subsequently, photomicrographs were taken at various time points.

### Transwell invasion assay

Chambers with polycarbonate filters with a 8 μm nominal pore size (Millipore, Boston, MA) coated on the upper side with Matrigel (BD Bioscience, San Jose, CA) were used to assess cell invasiveness. The chambers were placed into a 24-well plate. Cells were trypsinized, resuspended in serum-free medium, and seeded at a density of 1 × 10^5^ cells per well in the upper chambers. The lower chambers were filled with 500 μl RPMI-1640 containing 10% FBS. After 24 h, the chambers were moved to a fresh 24-well plate and stained with 0.2% crystal violet for 20 min. The number of cells that had attached to the lower surface was counted under a light microscope and statistically analyzed.

### RNA interference assay

Cells were transfected with siRNAs using Lipofectamine 2000 (Invitrogen, Carlsbad, CA, USA) according to the manufacturer’s instructions. siRNAs targeting NEK6 were designed and synthesized by Shanghai GenePharma Co. (Shanghai, China). The siRNA sequences used are depicted in Table S1.

### Apoptosis and cell cycle assays

The apoptosis and cell cycle rates of Huh-7 CD147-KO cells transfected with wildtype CD147 or S246A/S252A mutants were assessed using an Annexin V-FITC/propidium iodide (PI) apoptosis detection kit and a cell cycle detection kit, respectively (KeyGEN Biotech, Nanjing, China). Quantification of PI and FITC signals was performed using a fluorescence activated cell sorter FACSAria (BD Bioscience, San Jose, CA) system.

### qRT-PCR

Total RNA was extracted using TRIzol reagents (OMEGA Bio-Tek, Norcross, GA, USA). Reverse transcription was performed using a PrimeScript RT reagent kit (TaKaRa Biotechnology, Japan). All primers were synthesized by BGI (BGI, Shenzhen, China) and their sequences are listed in Table S1. Quantitative real-time PCR (qRT-PCR) was performed using a SYBR Premix Ex Taq II Kit (TaKaRa Biotechnology, Japan).

### RNA sequencing

Total RNA was extracted using Trizol (Tiangen, Beijing) and assessed for quantity and quality using an Agilent 2100 BioAnalyzer (Agilent Technologies, Santa Clara, CA, USA) and a Qubit Fluorometer (Invitrogen, Carlsbad, CA, USA), respectively. Total RNA samples that met the following requirements were used in subsequent experiments: RNA integrity number (RIN) > 7.0 and a 28S:18S ratio > 1.8. Sequence libraries were generated and sequenced by CapitalBio Technology (Beijing, China). A NEB Next Ultra RNA Library Prep Kit for Illumina (NEB, Ipswich, MA, USA) was used to construct the libraries for sequencing. A NEB Next Poly(A) mRNA Magnetic Isolation Module (NEB, Ipswich, MA, USA) kit was used to enrich poly(A) tailed mRNA molecules from 1 μg total RNA. The mRNA was fragmented into ∼200 base pair pieces. First-strand cDNA was synthesized from the mRNA fragments using reverse transcriptase and random hexamer primers, after which second-strand cDNA was synthesized using DNA polymerase I and RNase H. The ends of the cDNA fragments were subjected to an end repair process including the addition of a single “A” base, followed by ligation of adapters. The resulting products were purified and enriched by PCR to amplify the library DNA. The final libraries were quantified using a KAPA Library Quantification kit (KAPA Biosystems, South Africa) and an Agilent 2100 Bioanalyzer. After qRT-PCR validation, the libraries were subjected to paired-end sequencing with a pair-end 150-base pair reading length on an Illumina HiSeq sequencer (Illumina) [30].

The sequencing quality was assessed using FastQC (Version 0.11.5) after which low quality data were filtered using NGSQC (v0.4). The clean reads were subsequently aligned to the reference genome using HISAT2 (Johns Hopkins University, USA) with default parameters [31]. The processed reads from each sample were aligned using HISAT (Johns Hopkins University, USA) against the corresponding human reference genome. Gene expression analyses were performed using Cuffquant and Cuffnorm (Cufflinks 2.2.1). Cuffdiff was used to analyze the differentially expressed genes (DEGs) between samples. The standardization method of Cuffdiff is geometric, with per-condition and pooled as discrete model [32]. Thousands independent statistical hypothesis-driven tests were conducted on DEGs, separately, after which a *p* value was obtained that was corrected using a FDR method. This *p* value was used to perform significance analysis. The parameters for classifying significantly DEGs was ≥ 2-fold difference (|log2FC| ≥ 1, FC: fold change of expression) in transcript abundance. By searching the ENSEMBL, NCBI, UniProt, GO and KEGG databases, BLAST (Basic Local Alignment Search Tool) alignment was performed to determine the functional annotation of the DEGs. The best matches were selected to annotate the DEGs. Finally, a KEGG pathway enrichment analysis was performed for the DEGs using KOBAS 3.0 software (Available online: http://kobas.cbi.pku.edu.cn).

### Statistical analysis

All experiments were performed in triplicate, and the results were expressed as mean ± SD. Statistics were evaluated using GraphPad Prism V7.0 software (GraphPad Software, La Jolla, CA). The statistical analyses were carried out using one-way ANOVA (multiple comparisons) and Student’s t test (two comparisons). Differences were deemed significant when *p* < 0.05. *** indicates *p* < 0.001, ** indicates *p* < 0.01, * indicates *p* < 0.05, and # indicates *p* > 0.05.

## Results

### CD147 is phosphorylated in primary HCC tissues and derived cell lines

**Fig. 1:**
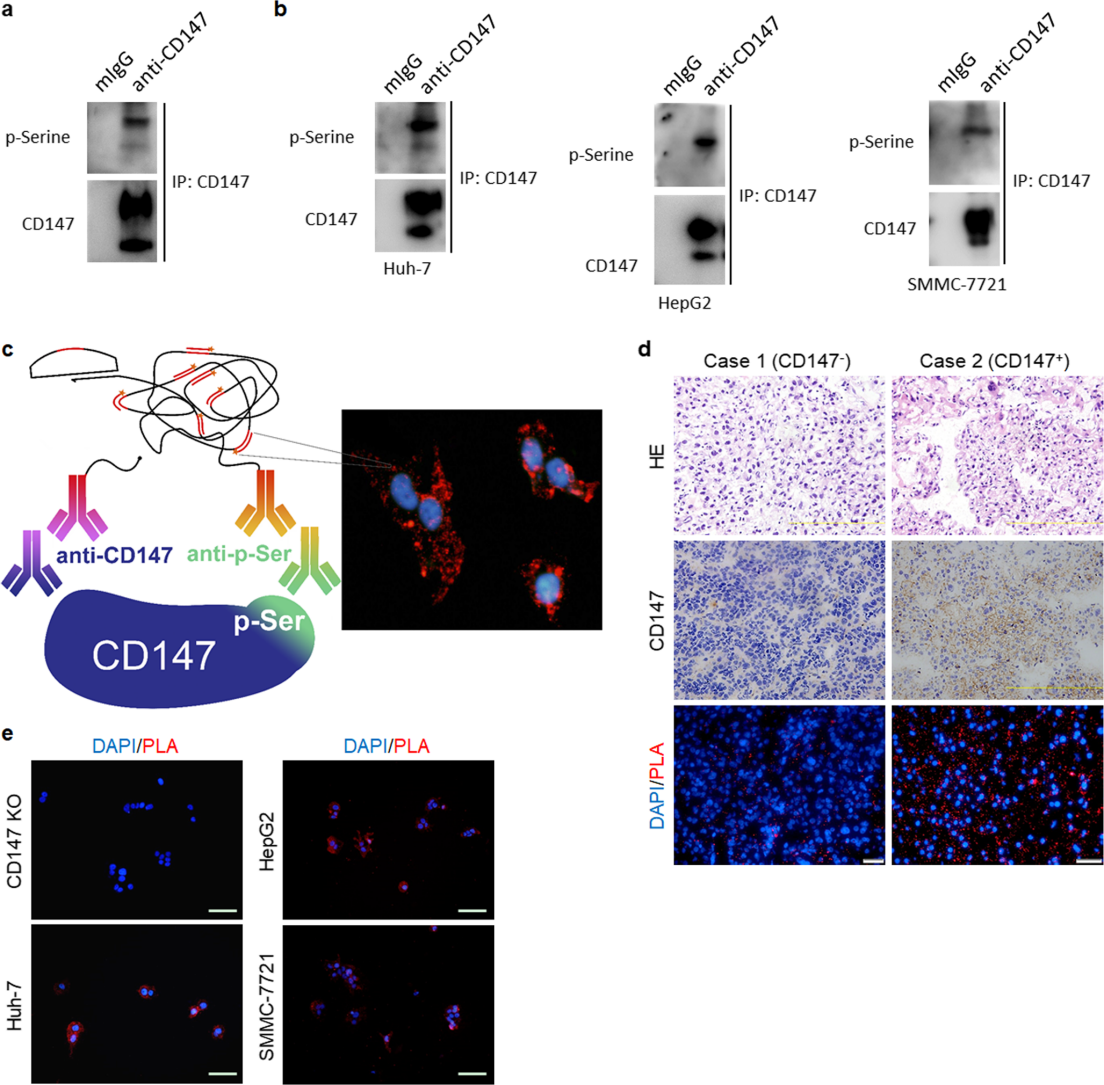
**CD147 is phosphorylated in HCC tissue and cell lines. a-b** CD147 was immunoprecipitated from lysates of HCC tissue (**a**) and cell lines (**b**) using a monoclonal antibody directed against the ectodomain of CD147, whereas phosphorylation modification was detected using an anti-phospho-serine antibody. **c** Schematic depiction of in situ PLA-based detection of CD147 phosphorylation modification. **d** Two representative HCC specimens stained with HE (top panels), immuno-stained for CD147 (middle panels) and in situ PLA stained for CD147 phosphorylation modification (bottom panels). Scale bar, 100 μm in IHC and HE, 20 μm in PLA. **e** In situ PLA-based detection of CD147 phosphorylation modification in indicated HCC cells. Scale bars, 20 μm

As mass spectrometry-based global phosphoproteome analysis has revealed that CD147 can be phosphorylated at Ser246 and/or Ser252 in various human tissues and cell lines, we first examined whether CD147 is also phosphorylated in HCC tissues and cell lines. Total CD147 was purified from HCC tissue and cell lysates by immunoprecipitation after which phosphorylation modification was detected using an anti-phospho-serine antibody. We found that CD147 was phosphorylated in HCC tissues (Fig. 1a) and Huh-7, HepG2 and SMMC-7721 cells (Fig. 1b), whereas no positive result was obtained using the anti-phospho-serine antibody in a mouse IgG (mIgG) isotype control group. To characterize CD147 phosphorylation in situ, we applied a PLA assay, which detects post-translation modifications (PTMs) including phosphorylation in situ in fixed biological samples employing two paired antibodies, i.e., an anti-phospho-serine antibody and an anti-CD147 antibody (Fig. 1c) [33, 34]. The validity of this approach was established by monitoring CD147 phosphorylation in FFPE HCC specimens. As a negative control, we analyzed a HCC tissue without CD147 expression as verified by IHC staining. Little background staining was observed, whereas distinct red spots were detected in a HCC tissue with positive CD147 expression (Fig. 1d). We also detected a similar pattern of red spots in Huh-7, HepG2 and SMMC-7721 cells, whereas little staining was observed in Huh-7 CD147-KO cells (Fig. 1e). Together, these results indicate that CD147 is serine phosphorylated in HCC tissues and cell lines.

### CD147 is phosphorylated at S246 and S252 in HCC tissues and cell lines

**Fig. 2:**
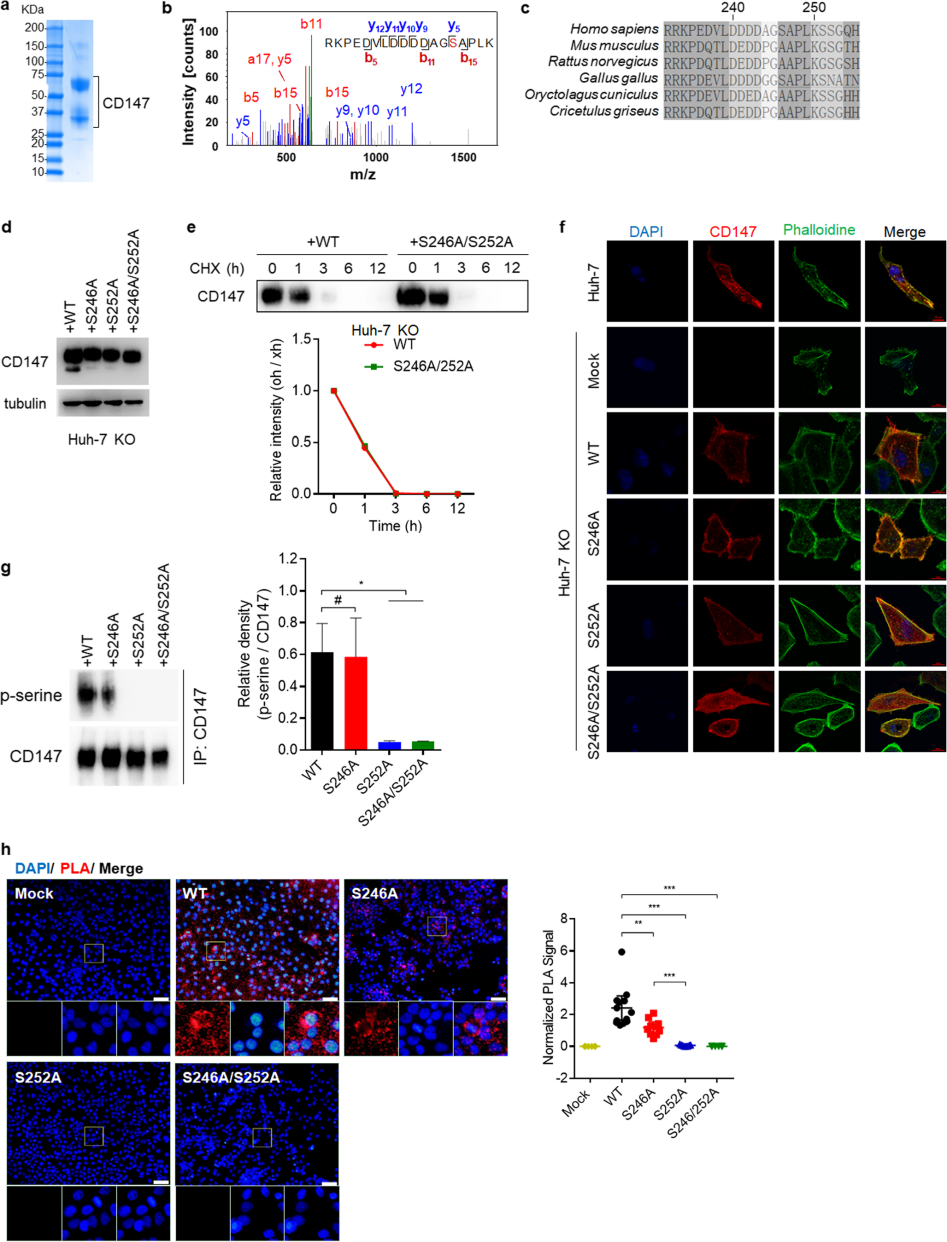
**Identification of CD147 phosphorylation sites in HCC tissue and cell lines. a** Coomassie brilliant blue staining of native CD147 purified from HCC tissue. The molecular weights for protein standards (left lane) are indicated. **b** Tandem mass spectrometry of precursor ions in the phosphorylated CD147 peptide (amino acids 233–250; sequence RKPEDVLDDDDAGSAPLK, where S (red) indicates phosphorylated serine). **c** C-terminal amino acid sequence alignment of human, mouse, rat, chicken, rabbit and Chinese hamster CD147 residues. **d** Western blot analysis of lysates from Huh-7 KO cells transfected with various constructs as indicated. **e** Huh-7 KO cells transfected with indicated constructs and treated with cycloheximide (CHX). Cells were harvested at various time points and evaluated by Western blotting for CD147 expression. The intensity of each band was measured and normalized so that the intensity at time = 0 was 1. **f** Huh-7 and Huh-7 KO cells transfected with indicated constructs were fixed, after which the distribution of CD147 and microfilaments was determined using immunofluorescence staining and confocal laser scanning microscopy. Scale bars, 10 μm. **g** CD147 was immunoprecipitated from lysates of Huh-7 KO cells transfected with indicated constructs after which phosphorylation modification was detected using an anti-phospho-serine antibody. The relative phosphorylation levels of CD147 are reflected by the ratio of phospho-CD147 optical density values to those of total CD147. * *p* < 0.05, # *p* > 0.05 by unpaired t test. **h** In situ PLA-based detection of CD147 phosphorylation modification in Huh-7 KO cells transfected with the indicated constructs. PLA signals per cell are shown in a diagram. Bars represent averages with 95% confidence interval. Scale bars, 20 μm. ** *p* < 0.01, *** *p* < 0.001 by unpaired t test

Based on the observation that CD147 is serine phosphorylated in HCC tissues, we next set out to identify the precise phosphorylation site of CD147 in HCC tissue. Native CD147 was isolated from HCC tissue using an anti-CD147 antibody (Fig. 2a). The isolated CD147 protein was subsequently subjected to mass spectrometry. By doing so, an approximately 80 Da mass shift was observed on serine-246, suggesting that CD147 is phosphorylated at this serine residue in HCC tissue (Fig. 2b). Previous studies showed that CD147 may be phosphorylated at serine-246 and/or serine-252 in different tissues, and sequence analysis has revealed that serine-246 and serine-252 are evolutionarily conserved in CD147 in species ranging from human, mouse, rat, chicken, rabbit and Chinese hamster (Fig. 2c). Next, we set out to evaluate the phosphorylation level of each of these serines in Huh-7 cells. To this end, we constructed S246A, S252A and S246A/S252A mutants of CD147 and assessed their steady-state expression in Huh-7 CD147 KO cells (Fig. 2d). We found that wild type CD147 and S246A/S252A mutants exhibited similar turnover rates at the same time (Fig. 2e) and that the mutations did not alter the membrane distribution pattern of CD147 (Fig. 2f). Next, we analyzed the phosphorylation level of each mutant by immunoprecipitation using an anti-phospho-serine antibody. We fount that wildtype CD147 exhibited a higher level of phosphorylation modification than the mutants and that no phosphorylation of S252A and the S246A/S252A mutants could be detected (Fig. 2g). To further confirm the above results, In situ Duolink-PLA analysis was performed. As a negative control, we found that the PLA signal of Huh-7 CD147-KO cells was below the detection threshold. Compared to wild type, the PLA signal of S246A phosphorylation was found to be reduced, while the PLA signals of S252A and S246A/S252A were found to be low (Fig. 2h). Taken together, these results indicate that the major phosphorylation sites of CD147 are serine-246 and serine-252 in HCC tissues and cells.

### Non-phosphorylated CD147 promotes HCC cell migration and invasion

**Fig. 3:**
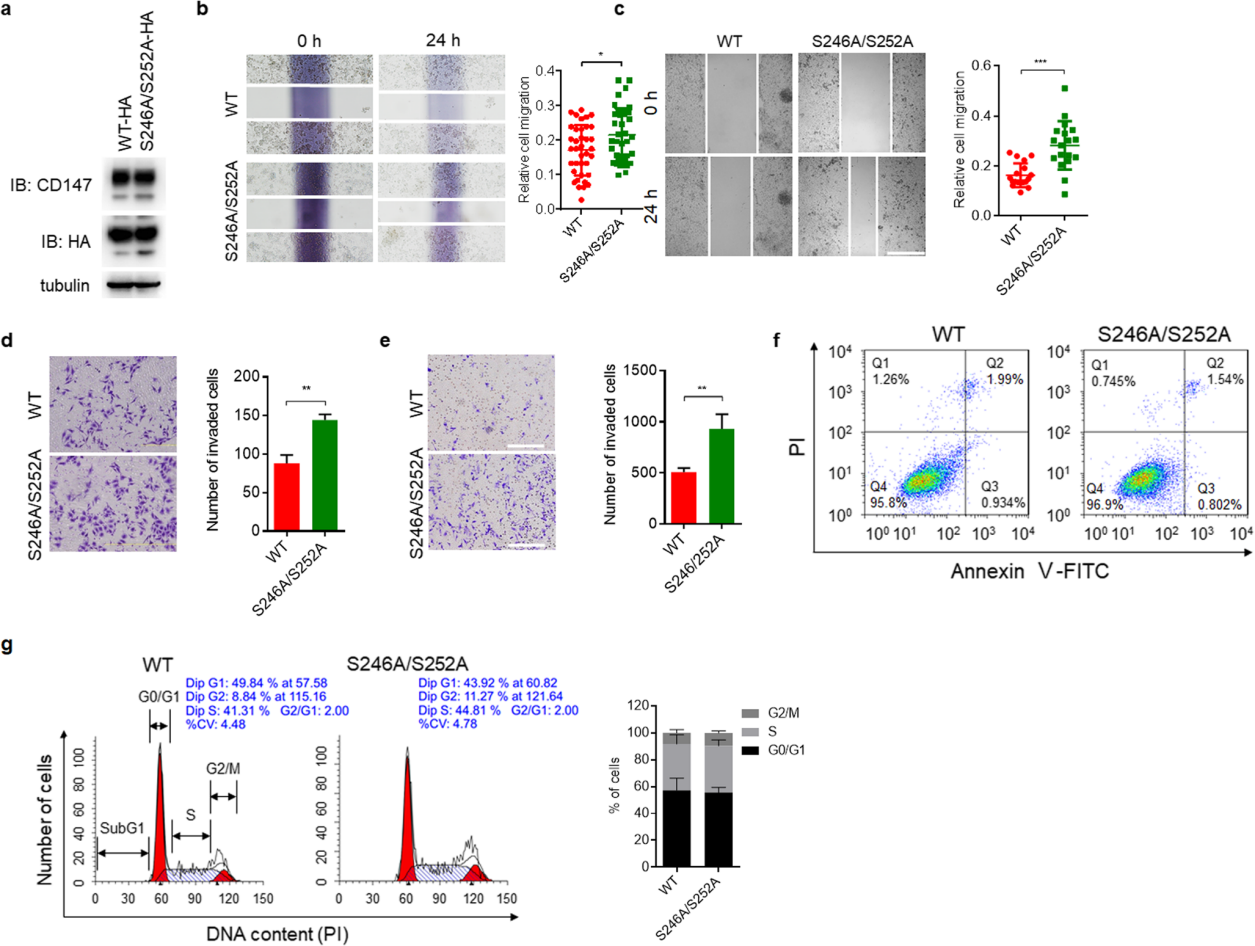
**Hypo-phosphorylated CD147 promotes HCC cell migration and invasion. a** Western blot analysis of HepG2 shCD147 cells transfected with various constructs as indicated. **b-c** Representative images of scratch wound healing assays using HepG2 shCD147 cells (**b**) or Huh-7 KO cells (**c**) transfected with wildtype CD147 or S246A/S252A mutant. Scale bar, 100 μm. Diagrams show quantitative analyses of relative migration distances. **d-e** Representative images of transwell invasion assays using HepG2 shCD147 cells (**d**) or Huh-7 KO cells (**e**) transfected with wildtype CD147 or S246A/S252A mutant. Scale bar, 100 μm. Graph shows quantitative analysis of the number of invaded cells per field. **f** Representative images of Annexin V-FITC and PI staining using Huh-7 KO cells transfected with wildtype CD147 or S246A/S252A mutant. **g** Flow cytometry analysis showing cell cycle progression of Huh-7 KO cells transfected with wildtype CD147 or S246A/S252A mutant. Graph shows cell cycle distribution. * *p* < 0.05, ** *p* < 0.01, *** *p* < 0.001 by unpaired t test

To determine the biological significance of CD147 phosphorylation, we transfected Huh-7 CD147-KO (Fig. 2d) and HepG2 shCD147 (Fig. 3a) cells with S246A/S252A mutant or wildtype CD147. Subsequent cell migration abilities were determined using scratch wound healing assays. We found that CD147 expression rescue of the S246A/S252A mutants significantly increased the migration capacity of both HepG2 shCD147 (Fig. 3b) and Huh-7 CD147-KO (Fig. 3c) cells compared to the respective wildtype controls. Consistently, transwell invasion assays showed that S246A/S252A mutants enhanced cell invasion compared to the wildtype control in both HepG2 shCD147 (Fig. 3d) and Huh-7 CD147-KO cells (Fig. 3e). These results indicate that CD147-facilitated migration and invasion of HCC cells are incompatible with phosphorylation events. As CD147 was reported to be able to regulate cytoskeleton rearrangements, we assessed the effects of CD147 phosphorylation on the cytoskeleton in HCC cells. We found that CD147 mutants as well as wildtype CD147 were mainly located in the cell membrane, and no significant alterations in the cytoskeleton were observed (Fig. 2f). We also determined the effects of CD147 phosphorylation on the occurrence of cell death and cell cycle progression. We found that HCC cells expressing S246A/S252A mutants showed similar apoptotic cell proportions (Fig. 3f) and cell cycle progression parameters (Fig. 3g) compared to the wildtype controls, suggesting that phosphorylation modification of CD147 is not involved in the regulation of CD147-mediated cytoskeleton rearrangements, apoptosis and/or cell cycle progression.

### Hypo-phosphorylated CD147 enhances STAT3 and Akt signaling and alters the expression of genes involved in ECM remodeling

**Fig. 4:**
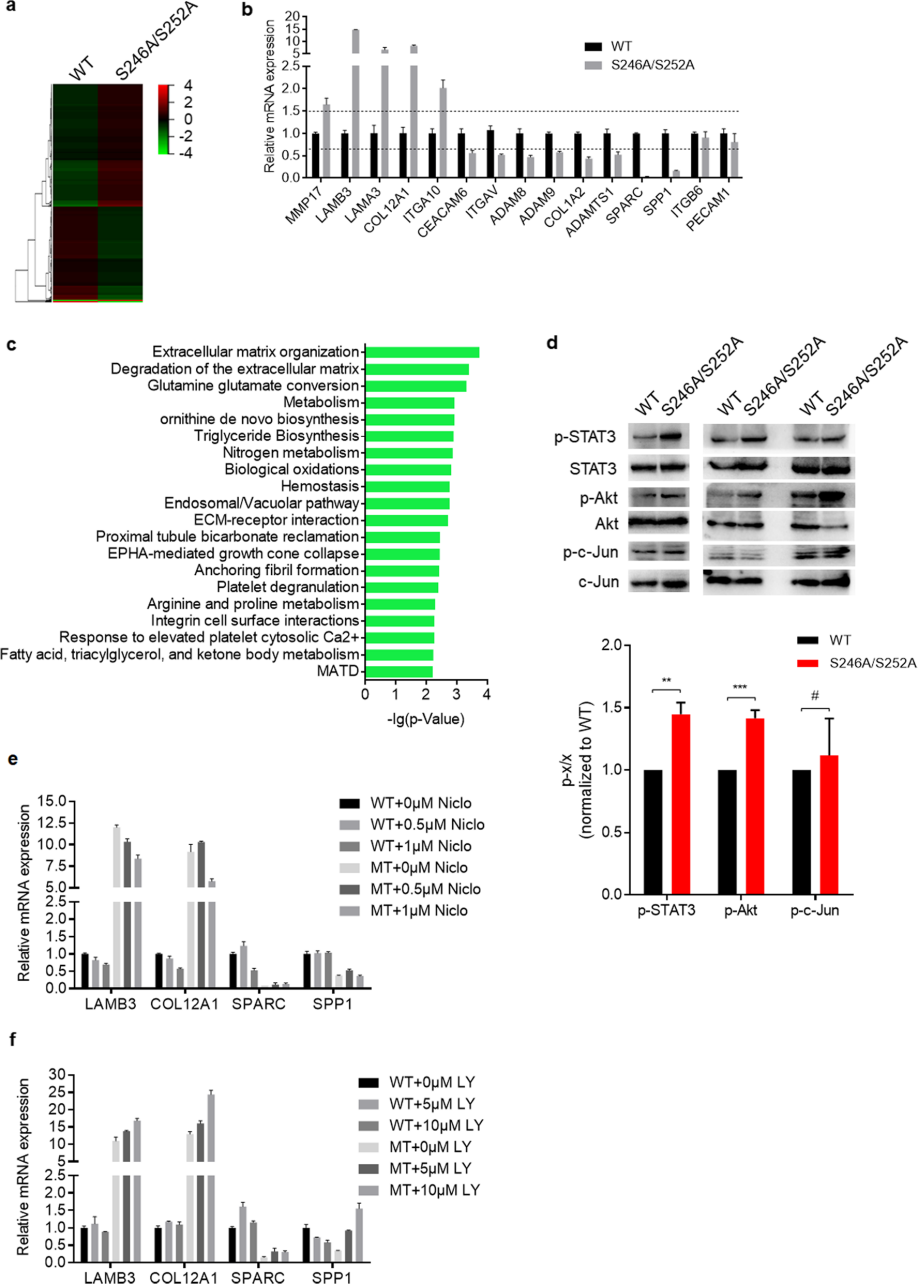
**Hypo-phosphorylated CD147 alters the expression of ECM remodeling-related genes and enhances STAT3 and Akt signaling. a** Genome-wide analysis of mRNA expression changes in Huh-7 KO cells transfected with wildtype CD147 or S246A/S252A mutant. **b** Validation of differentially expressed ECM remodeling-related genes using qRT-PCR. **c** Column chart of the top 20 KEGG enrichments. **d** Western blot analysis of the indicated proteins in Huh-7 KO cells transfected with WT CD147 or S246A/S252A mutant. The relative phosphorylation levels of STAT3 and Akt were measured and normalized so that the phosphorylation level in wild type cells was 1. # *p* > 0.05, ** *p* < 0.01, *** *p* < 0.001 by unpaired t test. **e-f** Relative qRT-PCR analysis of the indicated genes in Huh-7 KO cells transfected with wildtype CD147 or S246A/S252A mutant. Cells were treated with STAT3 inhibitor Niclocide (Nico) (**e**) or Akt inhibitor LY294002 (LY) (**f**)

In order to explore the mechanisms by which non-phosphorylated CD147 promotes HCC cell migration and invasion, we employed gene expression profiling in Huh-7 KO cells transfected with S246A/S252A mutant CD147 or wildtype CD147. Of the 632 differentially expressed genes identified, 358 genes were up-regulated and 274 genes were down-regulated (Fig. 4a). Fifteen of the differentially expressed genes (DEGs) were validated by qRT-PCR (Fig. 4b). Subsequent KEGG pathway analysis showed that ECM organization and ECM degradation were the top two pathways involved (Fig. 4c), which corroborates the importance of CD147 in ECM remodeling and subsequent invasion during tumor progression. Since CD147 has been reported to regulate several signaling pathways, such as the STAT3 [35, 36], Akt [37, 38] and c-Jun [39] pathways, we wondered whether this regulation may be dependent on the phosphorylation state of CD147. We found that, in contrast to wildtype CD147, expression of S246A/S252A mutant CD147 significantly increased the STAT3 and Akt phosphorylation levels (Fig. 4d), suggesting that CD147 promotes STAT3 and Akt signaling mainly through its non-phosphorylated form. To ascertain whether the altered gene expression profiles observed were due to activated STAT3/Akt signaling, Huh-7 CD147-KO cells transfected with S246A/S252A mutant CD147 or wildtype CD147 were treated with STAT3 inhibitor Niclocide or Akt inhibitor LY294002. We found that the S246A/S252A mutant-induced upregulation of LABM3 and COL12A1 could be partially reversed by inhibition of STAT3 in a dose‐dependent manner (Fig. 4e). Consistently, we found that S246A/S252A mutant-induced downregulation of SPP1 could be reversed by inhibition of Akt in a dose-dependent manner (Fig. 4f). These results suggest that other pathways besides the STAT3/Akt signaling pathways may be responsible for non-phosphorylated CD147-induced alterations in gene expression in HCC cells.

### Hypo-phosphorylated CD147 enhanced cell migration and invasion can be reversed by STAT3 and Akt inhibitors

**Fig. 5:**
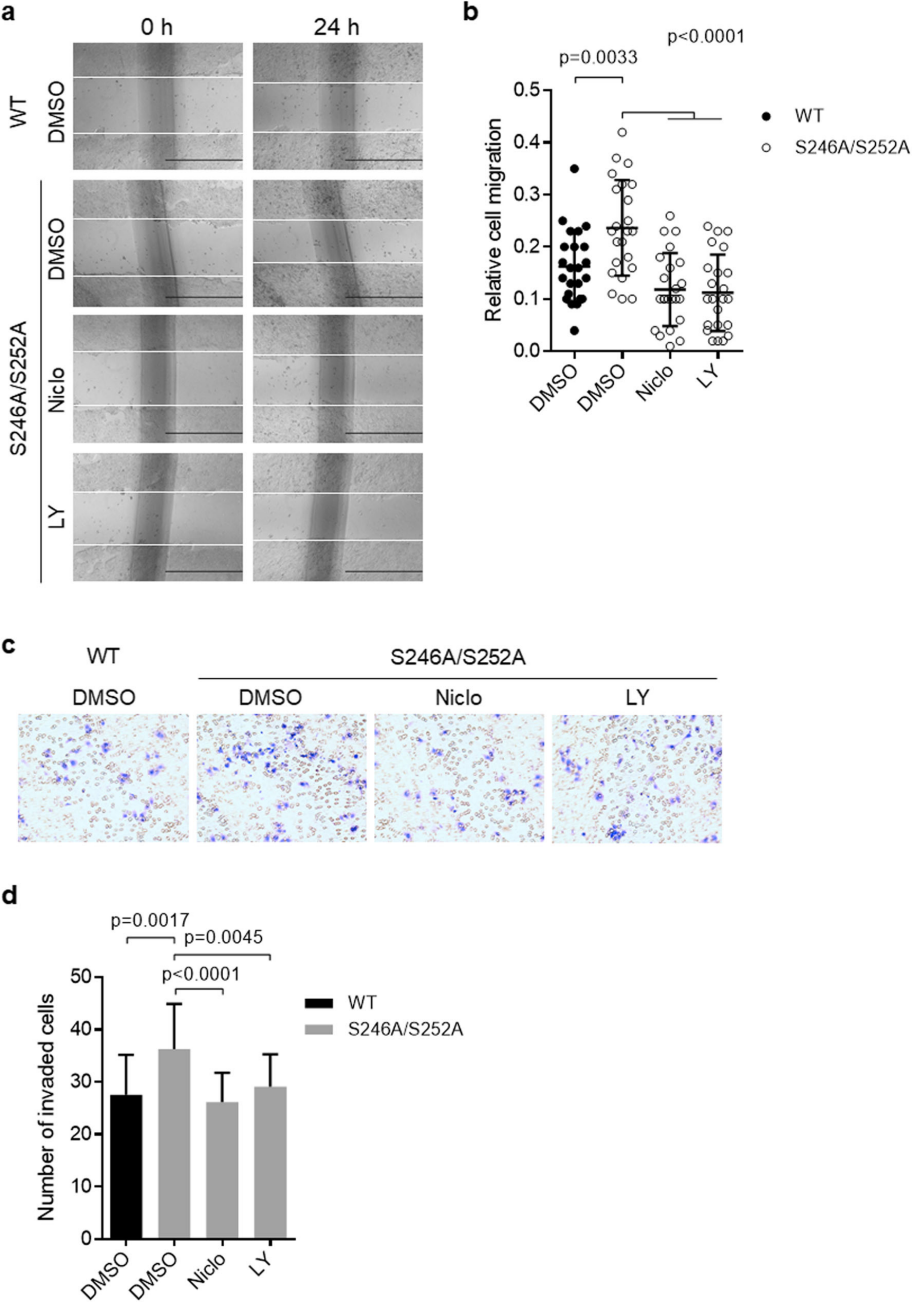
**Hypo-phosphorylated CD147 enhanced cell migration and invasion can be reversed by STAT3 and Akt inhibition. a** Representative images of scratch wound healing assays using Huh-7 CD147-KO cells transfected with wildtype CD147 or S246A/S252A mutant. Cells were treated with Niclocide (Nico) or LY294002 (LY). Scale bar, 100 μm. **b** Diagram showing quantitative analyses of relative migration distances. **c** Representative images of transwell invasion assays using Huh-7 CD147-KO cells transfected with wildtype CD147 or S246A/S252A mutant. Cells were treated with Niclocide (Nico) or LY294002 (LY). Scale bar, 100 μm. **d** Graph showing quantitative analyses of the number of invaded cells per field. The *p* values are derived from unpaired t test

As we found that S246A/S252A can promote HCC cell migration and invasion and increase STAT3 and Akt activation, we wondered whether STAT3 and Akt signaling are involved in hypo-phosphorylated CD147-induced cell migration and invasion. When Huh-7 CD147-KO cells expressing S246A/S252A mutant CD147 were treated with the STAT3 inhibitor Niclocide or the Akt inhibitor LY294002, we found that the S246A/S252A mutants promoted cell migration compared to the wild type group and that this effect could be abolished by both the STAT3 and Akt inhibitors (Fig. 5a and Fig. 5b). Consistently, we found that Huh-7 CD147-KO cells expressing S246A/S252A mutants showed enhanced invasive abilities compared to the wild type group and that these enhanced abilities could be abolished by both the STAT3 and Akt inhibitors (Fig. 5c and Fig. 5d). Together, these results suggest that S246A/S252A mutant CD147 can promote cell migration and invasion, at least partially through STAT3 and Akt signaling.

### NEK6 promotes and CyPA inhibits CD147 phosphorylation in HCC cells

**Fig. 6:**
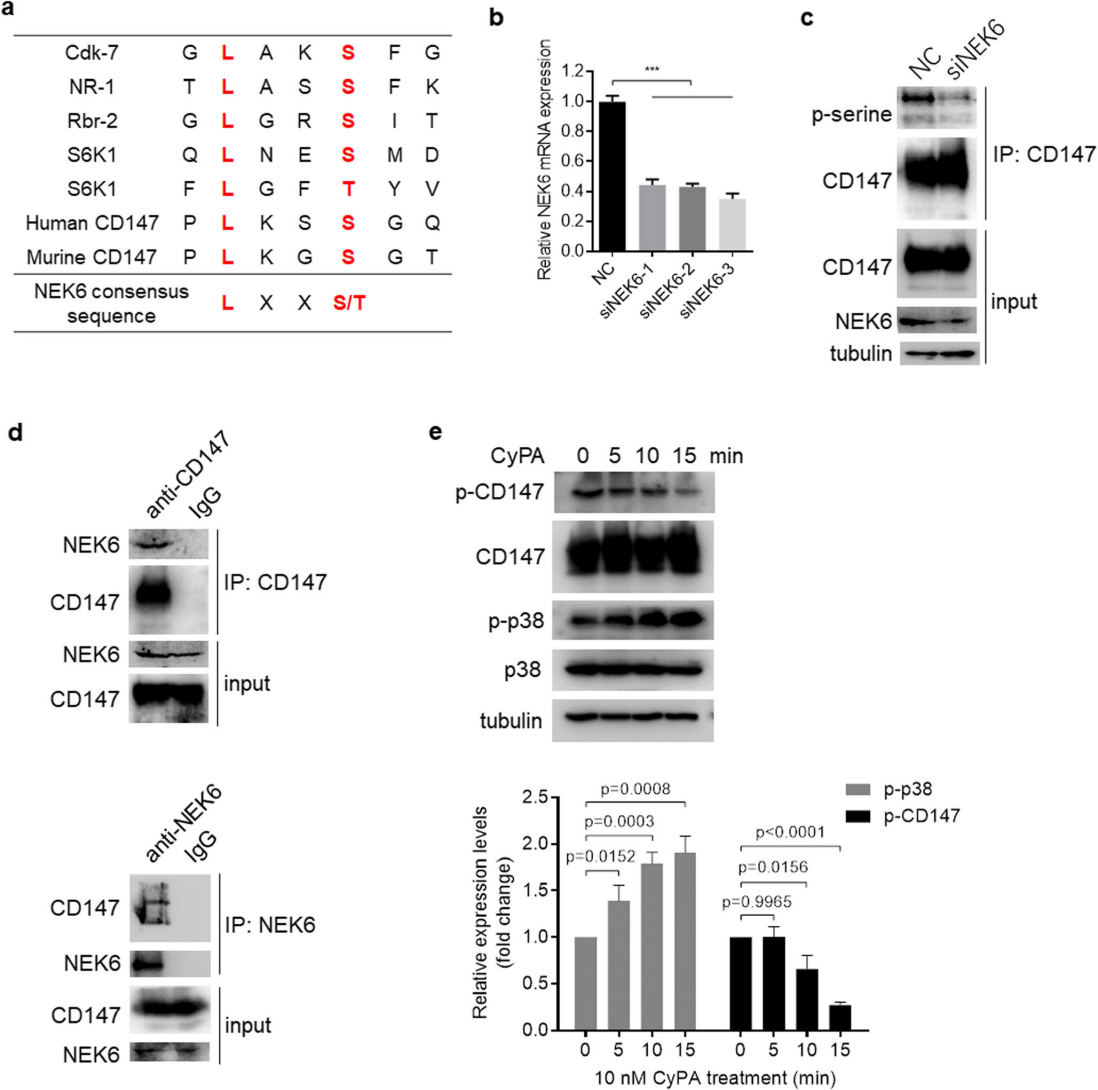
**NEK6 promotes and CyPA inhibits CD147 phosphorylation in HCC cells. a** Consensus sequence for NEK6 substrates. Sequences for CD147 and other known NEK6 substrates are also shown for comparison. **b** Relative qRT-PCR expression analysis of NEK6 in Huh-7 cells transfected with negative control siRNA or siRNAs targeting NEK6. **c** CD147 was immunoprecipitated from lysates of Huh-7 cells using a monoclonal antibody directed against the ectodomain of CD147 whereas its phosphorylation modification was detected using an anti-phospho-serine antibody. Cells were transfected with control siRNA (NC) or siRNAs targeting NEK6 (siNEK6). **d** Western blot analyses of endogenous CD147 co-IP with endogenous NEK6. IgG was used as a control antibody for immunoprecipitation. **e** Western blot analyses of the indicated proteins in Huh-7 cells treated with 10 nM CyPA for indicated time periods. The relative phosphorylation level for p38 and CD147 at the indicated time points was measured and normalized so that the phosphorylation level at time = 0 was 1. The *p* values are derived from unpaired t test

Next, we set out to assess whether CD147 phosphorylation is regulated by and involved in signal transduction. Serine-252 is located in a conserved sequence APLKSSGQ within human CD147, or APLKGSGT within murine CD147. The peptide matches the typical consensus motif (L-X-X-S/T) (Fig. 6a) [40], which is also found in TPP1 that is known to be phosphorylated by NEK6 [41]. Based on this information, we focused on the involvement of NEK6 as a possible kinase responsible for phosphorylating CD147. To this end, we transfected Huh-7 cells with siRNAs targeting NEK6 (Fig. 6b). We found that siRNA-mediated silencing of NEK6 decreased CD147 phosphorylation (Fig. 6c). To subsequently test a direct interaction between CD147 and NEK6, reciprocal co-IP was performed in Huh-7 cells. By doing so, we found that NEK6 was specifically immunopurified with CD147 (Fig. 6d). These results indicate that NEK6 directly interacts with CD147 and phosphorylates the protein at serine-252 in Huh-7 cells.

Since CD147 has been found to act as a cell surface signaling receptor for extracellular CyPA [42, 43] and to interact with the ectodomain of CD147 [44], we assessed a possible role of the intracellular domain of CD147 in signal transduction initiated by CyPA. To this end, we probed CD147 phosphorylation of cell lysates from Huh-7 cells stimulated with CyPA for different periods of time. After stimulation by CyPA, the phospho-CD147 band showed a specific down-shift after 10 min (Fig. 6e). In addition, phosphorylation of p38, known to be regulated by CyPA [45], was detected during the same time period. Based on these results, we conclude that serine-252 of CD147 is dephosphorylated after CyPA stimulation and that reversible phosphorylation of CD147 may be involved in signal transduction upon CyPA stimulation.

### Hypo-phosphorylation of CD147 is correlated with distal metastasis and poor overall survival in HCC patients

**Fig. 7:**
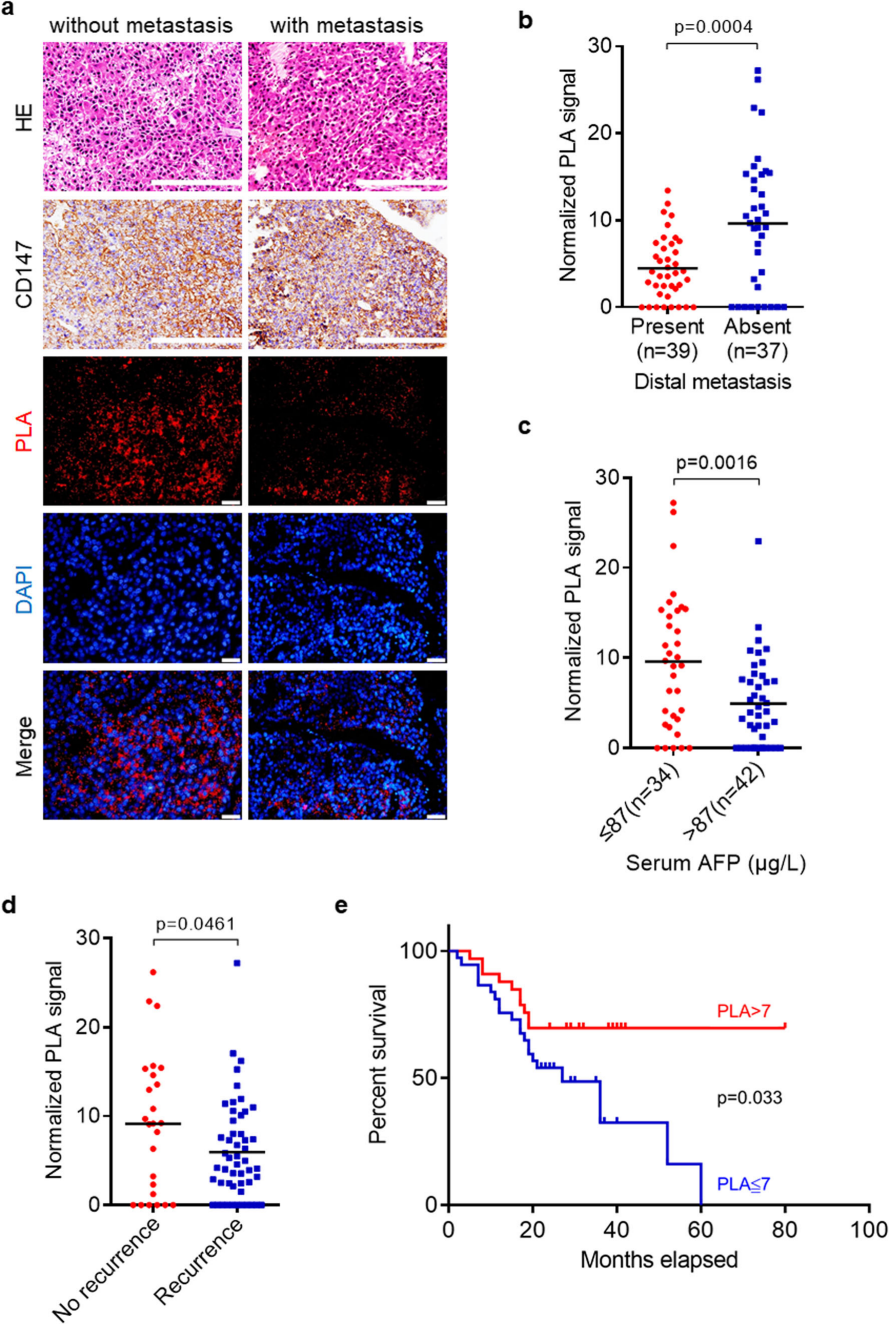
**Low phosphorylation level of CD147 correlates with distant metastasis and a poor survival of HCC patients. a** HCC tissues with (*n* = 39) or without (*n* = 37) distal metastases were examined for CD147 and phospho-CD147 levels using IHC staining and in situ PLA, respectively. Representative images of HE staining, IHC staining and in situ PLA are shown. Scale bar, 100 μm in IHC and HE, 20 μm in PLA. **b** Quantitative analysis of the levels of CD147 phosphorylation in HCC tissues with or without distal metastases. The PLA signals were counted and analyzed using Duolink ImageTool software. **c** Quantitative analysis of the levels of CD147 phosphorylation by in situ PLA analysis in HCC tissues grouped according to serum AFP level. **d** Quantitative analysis of the levels of CD147 phosphorylation by in situ PLA in HCC tissues with or without recurrence. **e** Kaplan-Meier analysis of overall survival for 70 patients based on levels of CD147 phosphorylation obtained by in situ PLA analysis. The *p* values are derived from unpaired t test (**b-d**) and Log-rank (Mantel-Cox) test (**e**)

Since we found that hypo-phosphorylated CD147 increases cell migration and invasion, we sought to determine whether CD147 phosphorylation is associated with the prognosis and clinical characteristics of HCC patients. To this end, we randomly sampled 76 tumor tissues from HCC patients with complete clinical and follow-up data (Table 1). Expression of CD147 was determined by IHC staining and CD147 phosphorylation was assessed by in situ PLA analysis (Fig. 7a). We found that the metastasis group had a lower PLA signal than the non-metastasis group (Fig. 7b), whereas no significant differences in the expression of CD147 between the two groups was noted (Table 2). The group with lower serum AFP levels (AFP ≤ 87 μg/l) exhibited higher PLA signals (Fig. 7c) and the recurrence group lower PLA signals compared to the group without recurrence (Fig. 7d). Pearson correlation analysis showed that high levels of phosphorylated CD47 expression were negatively correlated with serum AFP levels (*p* = 0.0016), distant metastasis (*p* = 0.0004) and recurrence (*p* = 0.0461), but were not correlated with age and/or any other factors tested (Table 1). Kaplan-Meier analysis showed that low PLA signals of CD147 phosphorylation were correlated with a poor overall survival (Fig. 7e). These results indicate that low CD147 phosphorylation levels correlate with the occurrence of distant metastasis and an unfavorable prognosis in HCC.

**Table 1:** Correlation between CD147 phosphorylation (PLA) and clinicopathological features of HCC patients

Features	PLA signal Mean (minimum-maximum)	*p* Value^a^
Gender		
Male (*n* = 63)	6.77 (0–27.20)	0.446
Female (*n* = 13)	8.16 (0–22.94)	
Liver cirrhosis		
Yes (*n* = 46)	6.13 (0–22.41)	0.393
No (*n* = 30)	8.35 (0–27.20)	
Portal vein metastasis		
Yes (*n* = 10)	4.83(0–15.35)	0.169
No (*n* = 66)	7.34(0–27.20)	
Number of tumors		
≤ 3 (*n* = 67)	7.27(0–27.20)	0.502
> 3 (*n* = 9)	5.14(0–10.98)	
Tumor size (cm)		
≤ 5 (*n* = 28)	6.58 (0–27.20)	0.363
> 5 (*n* = 48)	7.25 (0–26.20)	
Serum AFP (μg/l)		
≤ 87 (*n* = 34)	9.58 (0–27.20)	0.0016
> 87 (*n* = 42)	4.92 (0–22.94)	
Distal metastasis		
Yes (n = 39)	4.48 (0–13.42)	0.0004
No (n = 37)	9.67 (0–27.20)	
Recurrence		
Yes (*n* = 51)	5.96(0–27.20)	0.0461
No (*n* = 25)	9.14(0–26.20)	

^a^*p* values were calculated using Student’s t test. *p* values < 0.05 were considered to indicate statistical significance

**Table 2:** Correlation between CD147 expression and clinicopathological features of HCC patients

Features	CD147 expression				*p* Value^a^
	–	+	++	+++	
Gender					
Male (n = 63)	14	13	25	11	0.952
Female (n = 13)	3	2	5	3	
Liver cirrhosis					
Yes (n = 46)	10	11	18	7	0.636
No (n = 30)	7	4	12	7	
Portal vein metastasis					
Yes (n = 10)	5	1	4	0	0.086
No (n = 66)	12	14	26	14	
Number of tumors					
≤ 3 (n = 67)	15	13	27	12	0.976
> 3 (n = 9)	2	2	3	2	
Tumor size (cm)					
≤ 5 (n = 28)	8	8	8	4	0.235
> 5 (n = 48)	9	7	22	10	
Serum AFP (μg/l)					
≤ 87 (n = 34)	5	8	15	6	0.490
> 87 (n = 42)	12	7	15	8	
Distal metastasis					
Yes (n = 39)	8	8	15	8	0.949
No (n = 37)	9	7	15	6	
Recurrence					
Yes (n = 51)	11	12	20	8	0.614
No (n = 25)	6	3	10	6	

^a^*p* values were calculated using Pearson’s chi-squared test. *p* values < 0.05 were considered to indicate statistical significance

## Discussion

Phosphorylation is an important form of post-translational modification (PTM) [46]. Here, we uncovered a novel functional phospho-modification of the CD147 protein and confirmed its relevance for regulating its activity. Consistent with previous phospho-proteome work on other human tissues and cell lines, we show that CD147 may exhibit various degrees of phosphorylation in primary HCC tissues and derived cell lines, indicating that phosphorylation represents, next to glycosylation, a form of PTM for CD147 in these tissues and cells. Further analyses revealed that the level of phospho-CD147 in patients without metastases was higher than in those with distant metastases, indicating that low phospho-CD147 levels are relatively more potent in promoting metastasis in HCC.

CD147 is reported to exhibit two forms of PTM, glycosylation and phosphorylation. Mass spectrometry-based structural determination of N-glycans of CD147 purified from human lung cancer tissue has shown that native eukaryotic CD147 is N-glycosylated and contains a series of high-mannose and complex-type N-linked glycan structures. It has been found that glycosylated CD147 stimulates the secretion of MMPs more efficiently than non-glycosylated CD147 [47]. Mutation analysis revealed that glycosylation at asparagine-152 is important for proper CD147 protein folding in the ER and for its stability, thereby reinforcing HCC metastasis [48]. Here, we report that, in contrast to glycosylation, phosphorylation does not affect CD147 protein folding, nor its stability and/or translocation to the membrane. In addition, we found that phosphorylation acts as a dominant negative modification of CD147, which attenuates its promoting effects on HCC tumor cell migration and invasion, indicating that the non-phosphorylated form of CD147 is the active form that is related to tumor progression.

The transmembrane protein CD147 has a long extracellular domain and a short intracellular domain. Several studies have emphasized the critical role of the ectodomain of CD147 in promoting tumor cell proliferation, migration, invasion and metastasis, mainly by interacting with other transmembrane proteins such as integrins [49, 50], CD44 [51] and CD98 [52]. Previously, we found that both the ectodomain and intracellular domain of CD147 are required for mediating the effect of CD147 on the induction of MPPs and metastasis-related processes [53]. The potential effect of CD147 on metastasis-related processes such as adhesion and invasion in HCC cells was found to be abolished in cells expressing CD147 with a truncated C-terminal fragment. Here we provide evidence that removal of phosphorylation of the intracellular domain of CD147 enhances Akt and STAT3 signaling, which may contribute to accelerated HCC cell migration and invasion. Together, these results underscore the functional importance of the intracellular domain of CD147. The exact molecular mechanism by which the intracellular domain of CD147 affects HCC metastasis, however, still needs to be determined.

NEK6 is a serine/threonine kinase that has been identified as a homologue of the *Aspergillus nidulans* protein NIMA (never in mitosis A). NEK6 increases in abundance and activity during mitosis and its activation requires phosphorylation of Serine-206 at its activation loop [54]. Despite the critical role of NEK6 in maintaining proper anaphase progression [55, 56], the substrates of NEK6 have remained largely undefined. Here, we found that NEK6 interacts with and phosphorylates CD147. Accordingly, we found that NEK6 silencing leads to decreased CD147 phosphorylation in Huh-7 cells, indicating that CD147 is a substrate of NEK6. On the other hand we found that CyPA, a classical ligand for CD147, binds to its extracellular domain and, by doing so, reduces its phosphorylation. Whether CD147 phosphorylation is involved in NEK6 or CyPA regulated cell functions still needs to be determined, but the evidence presented here indicates that CD147 phosphorylation is regulatory and may be involved in downstream signal transduction.

In summary, our data provide evidence for a critical role of hypo-phosphorylated CD147 in HCC progression and imply that hypo-phosphorylated CD147 may serve as a valuable prognostic biomarker and as a target for the development of novel therapeutic modalities directed against HCC metastasis.

## Electronic supplementary material

ESM 1 (DOCX 17 kb)
