# Supplementary material for: Hypo-phosphorylated CD147 promotes migration and invasion of hepatocellular carcinoma cells and predicts a poor prognosis
Source: Cell Oncol (Dordr). 2019 Apr 23;42(4):537–54. doi: 10.1007/s13402-019-00444-0 (PMC12994331; doi:10.1007/s13402-019-00444-0)
Supplement: Supplementary file 1 — (DOCX 17 kb) [file 13402_2019_444_MOESM1_ESM.docx]

| Table S1 Oligonucleotide sequences of the primers and siRNAs | | | |
| --- | --- | --- | --- |
| Primers | NEK6 | Forward | 5’-CATCCCAACACGCTGTCTTTT-3’ |
|  | NEK6 | Reverse | 5’-TACACCTCGCTGAACTGTCCT-3’ |
|  | MMP17 | Forward | 5’-CACTCATGTACTACGCCCTCA-3’ |
|  | MMP17 | Reverse | 5’-TGGAGAAGTCGATCTGGATGTC-3’ |
|  | LAMB3 | Forward | 5’-GCAGCCTCACAACTACTACAG-3’ |
|  | LAMB3 | Reverse | 5’-CCAGGTCTTACCGAAGTCTGA-3’ |
|  | LAMA3 | Forward | 5’-CACCGGGATATTTCGGGAATC-3’ |
|  | LAMA3 | Reverse | 5’-AGCTGTCGCAATCATCACATT-3’ |
|  | COL12A1 | Forward | 5’-AGCTGAGGCAGACATTGTGTT-3’ |
|  | COL12A1 | Reverse | 5’-CCTCCTTTGTACGGCAAGTTT-3’ |
|  | ITGA10 | Forward | 5’-AACATCACCCACGCCTATTCC-3’ |
|  | ITGA10 | Reverse | 5’-GTTGGTAGTCACCTAAGTGGC-3’ |
|  | CEACAM6 | Forward | 5’-TCAATGGGACGTTCCAGCAAT-3’ |
|  | CEACAM6 | Reverse | 5’-CACTCCAATCGTGATGCCGA-3’ |
|  | ITGAV | Forward | 5’-ATCTGTGAGGTCGAAACAGGA-3’ |
|  | ITGAV | Reverse | 5’-TGGAGCATACTCAACAGTCTTTG-3’ |
|  | ADAM8 | Forward | 5’-GAGGGTGAGCTACGTCCTTG-3’ |
|  | ADAM8 | Reverse | 5’-CAGCCGTATAGGTCTCTGTGT-3’ |
|  | ADAM9 | Forward | 5’-TCCATTGCTCTTAGCGACTGT-3’ |
|  | ADAM9 | Reverse | 5’-GGGGTTCAATCCCATAACTCG-3’ |
|  | COL1A2 | Forward | 5’-GTTGCTGCTTGCAGTAACCTT-3’ |
|  | COL1A2 | Reverse | 5’-AGGGCCAAGTCCAACTCCTT-3’ |
|  | ADAMTS1 | Forward | 5’-ACTGGAAGCATAAGAAAGAAGCG-3’ |
|  | ADAMTS1 | Reverse | 5’-AATTCTGCCATCGACTGGTCT-3’ |
|  | SPARC | Forward | 5’-TGAGGTATCTGTGGGAGCTAATC-3’ |
|  | SPARC | Reverse | 5’-CCTTGCCGTGTTTGCAGTG-3’ |
|  | SPP1 | Forward | 5’-CTCCATTGACTCGAACGACTC-3’ |
|  | SPP1 | Reverse | 5’-CAGGTCTGCGAAACTTCTTAGAT-3’ |
|  | ITGB6 | Forward | 5’-TCCATCTGGAGTTGGCGAAAG-3’ |
|  | ITGB6 | Reverse | 5’-TCTGTCTGCCTACACTGAGAG-3’ |
|  | PECAM1 | Forward | 5’-AACAGTGTTGACATGAAGAGCC-3’ |
|  | PECAM1 | Reverse | 5’-TGTAAAACAGCACGTCATCCTT-3’ |
|  | GAPDH | Forward | 5’-GCACCGTCAAGGCTGAGAAC-3’ |
|  | GAPDH | Reverse | 5’-TGGTGAAGACGCCAGTGGA-3’ |
|  | S246A | Forward | 5’-CAGGGGTGCAGCGCCGGCGTCGT-3’ |
|  | S246A | Reverse | 5’-acgacgccggcgctgcacccctg-3‘ |
|  | S252A | Forward | 5’-ctggtgctgcccggcgctcttcaggggt-3’ |
|  | S252A | Reverse | 5’-acccctgaagagcgccgggcagcaccag-3’ |
| siRNAs | siNEK6-1 | Forward | 5’-GCAACUGAACCACCCAAAUTT-3’ |
|  | siNEK6-1 | Reverse | 5’-AUUUGGGUGGUUCAGUUGCTT-3’ |
|  | siNEK6-2 | Forward | 5’-GGAAGUACUUUGUGCAGCUTT-3’ |
|  | siNEK6-2 | Reverse | 5’-AGCUGCACAAAGUACUUCCTT-3’ |
|  | siNEK6-3 | Forward | 5’-GCACUACUCCGAGAAGUUATT-3’ |
|  | siNEK6-3 | Reverse | 5’-UAACUUCUCGGAGUAGUGCTT-3’ |
|  | NC | Forward | 5’-UUCUCCGAACGUGUCACGUTT-3’ |
|  | NC | Reverse | 5’-ACGUGACACGUUCGGAGAATT-3’ |
